# Association of adverse childhood experiences with adulthood multiple sclerosis: A systematic review of observational studies

**DOI:** 10.1002/brb3.3024

**Published:** 2023-05-01

**Authors:** Syeda Tayyaba Rehan, Zayeema Khan, Syed Hasan Shuja, Afia Salman, Hassan ul Hussain, Muhammad Saif Abbasi, Sufyan Razak, Huzaifa Ahmad Cheema, Sarya Swed, Salim Surani

**Affiliations:** ^1^ Department of Medicine Dow University of Health Sciences Karachi Pakistan; ^2^ Department of Neurology King Edward Medical University Lahore Pakistan; ^3^ Faculty of Medicine Aleppo University Aleppo Syria; ^4^ Adjunct Clinical Professor of Medicine Texas A&M University College Station Texas USA

**Keywords:** abuse, autoimmune disorders, emotional, neurological disorder, physical, sexual

## Abstract

**Introduction:**

Adverse childhood experiences (ACEs) are proposed to increase the risk of developing multiple sclerosis (MS) later in life. This systematic review aimed to explore the correlation between ACEs and MS development, age of onset, quality of life in MS patients and MS relapse rates.

**Methods:**

We searched a total of six databases in June 2022 and retrieved the relevant studies. The population included adult (18+) individuals who either had been diagnosed or were at risk for developing MS and also had exposure to ACEs. Our primary outcomes include the risks of MS development, age of MS onset, and MS relapse rate in patients who were exposed to different types of ACEs.

**Results:**

A total of 11 studies were included in our review. A study reported that among 300 women diagnosed with MS, 71 (24%) reported a history of childhood abuse; moreover, with further research, it was concluded that ACEs were associated with the development of MS. Abuse that occurred 2–3 times per week was associated with an 18.81‐fold increased risk of having MS when compared to the unexposed sample. The relapse rate of MS was found to be substantially greater in severe cases of ACEs compared to individuals who did not report any ACEs.

**Conclusions:**

Results support a significant association between ACEs and the development of MS; individuals with a positive history of ACEs develop MS symptoms earlier. Moreover, the severity of ACEs is also linked with increased relapse rates of MS.

## Graphical abstract

In this report, we aimed to explore the correlation between ACEs and MS to resolve the discrepancies in the existing studies. The present review is the first one to assess the association between ACEs and the development of MS in adulthood. Our results support a significant association between ACEs and the development of MS; individuals with a positive history of ACEs develop MS symptoms earlier. Moreover, the severity of ACEs is also linked with increased relapse rates of MS.

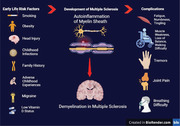

## 1 INTRODUCTION

**FIGURE 1:**
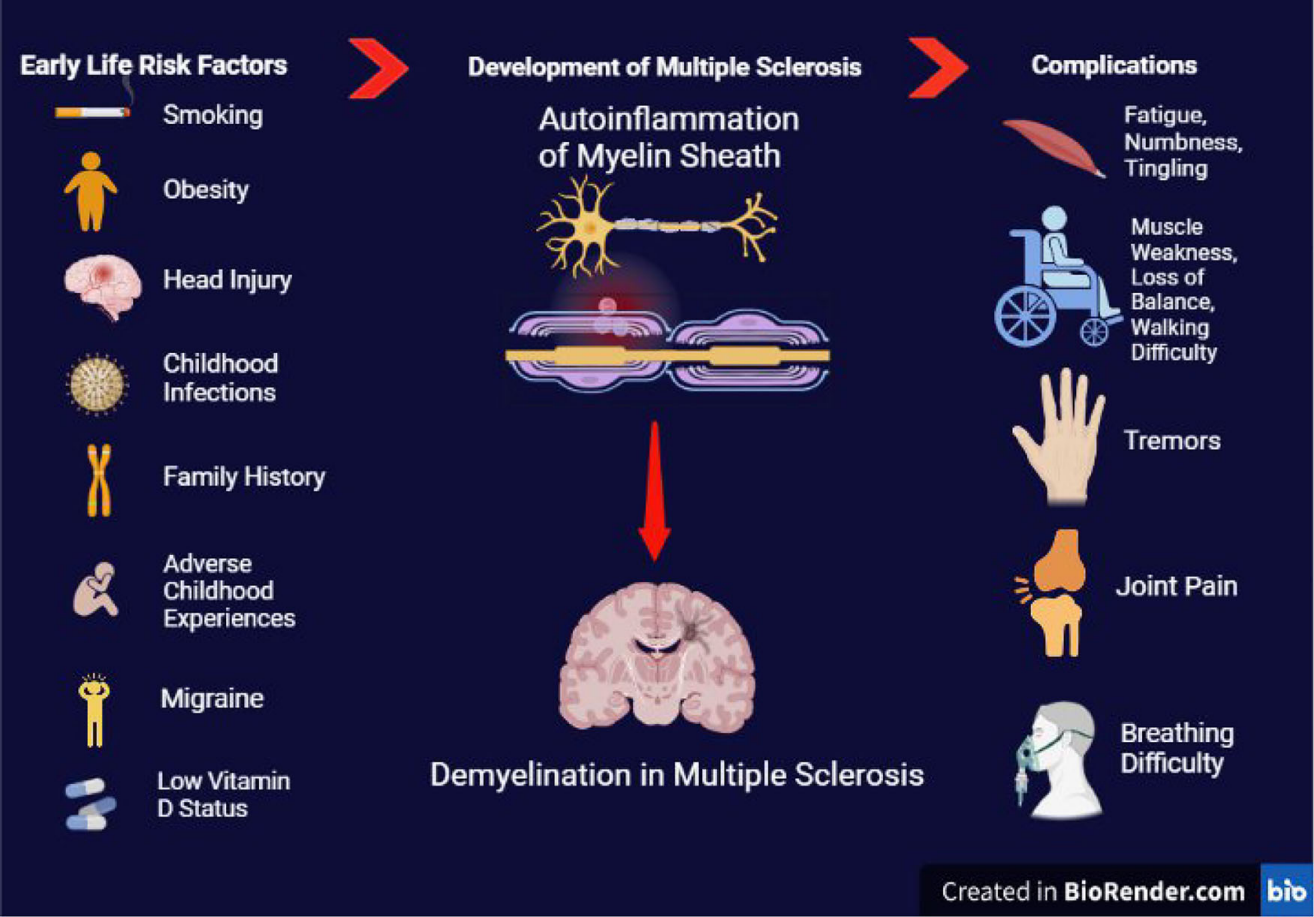
Risk factors of MS development, pathogenesis, and complications of disease (BioRender).

Multiple sclerosis (MS) is a chronic autoimmune disorder of the central nervous system (CNS) characterized by T‐cell‐mediated autoinflammation and demyelination of neurons with its typical presentation in young adulthood (Stadelmann et al., 2011). In 2016, an estimated 2.2 million people worldwide had MS, corresponding to a prevalence of 30.1 cases per 100,000 population (Wallin et al., 2019). In recent years, researchers have made significant progress in identifying the genetic and environmental factors associated with increased vulnerability to MS. However, the exact etiology of MS is yet to be identified (Handel et al., 2010; Shaw et al., 2017). Several early‐life exposures, such as pediatric optic neuritis (Waldman et al., 2011), low vitamin D status (Taan et al., 2021), frequent migraine (Taan et al., 2021), smoking (Taan et al., 2021), infections during childhood (Shaygannejad et al., 2016), and childhood obesity (Gianfrancesco et al., 2014), are recorded as predictors of late‐life MS development or discerned to be linked with several manifestations of adulthood MS (Figure 1).

Childhood trauma, abuse, neglect, and other forms of adverse childhood experiences (ACEs) lead to severe stress very early in a child's life, and individuals with these experiences are found to be at high risks for MS development in later life (Spitzer et al., 2012). ACEs or early childhood adversity refers to the wide variety of stressful situations that an individual may go through in childhood; these include childhood abuse, parental loss or divorce, and other forms of family dysfunctions (Kavanaugh et al., 2017; Nikulina & Widom, 2013; Ritchie et al., 2011). ACEs are associated with episodes of depression and anxiety, which may worsen and precipitate poor cognitive function, contributing to several disorders in adulthood (Kavanaugh et al., 2017; Nikulina & Widom, 2013; Ritchie et al., 2011).

Studies exploring the impact of stress on the brain have reported structural and functional alterations in prefrontal and limbic areas of the cerebrum in response to stress (McEwen, 2016; McEwen & Gianaros, 2011). Investigations further linked physical and mental stress to hyperactivation of the hypothalamic‐pituitary‐adrenal (HPA) axis, which may lead to dysregulation of immune response via increased secretion of corticotropin‐releasing factor (Heim et al., 2008). Several investigations also support a correlation between adverse childhood experiences and poorer health outcomes in adults, including the development of various heart diseases, stroke, and increased proinflammatory cytokines (Hepgul et al., 2012; Yang et al., 2013).

It is well established that an impaired immune system can lead to serious health conditions, particularly autoimmune disorders, which include MS, systemic lupus erythematosus, and rheumatoid arthritis. Since ACEs are also associated with immune dysregulation, increasing number of studies are supporting an association between ACEs and some autoimmune diseases (DeQuattro et al., 2020; Dube et al., 2009; Eilam‐Stock et al., 2021; Luiz et al., 2018). Many of the studies focus particularly on the association between ACEs and MS (Eilam‐Stock et al., 2021; Eid et al., 2022; Horton et al., 2022); however, the evidence relies substantially on cross‐sectional designs. Hence the present review aims to get an overview of all the published studies that interlink the ACEs to adult life MS.

Considering the prevalence of ACEs and MS around the world and their possible association, a systematic approach to reviewing data from multiple sources is imperative, and our study aims to address this knowledge gap. In this review, we aim to evaluate the association of adverse childhood experiences with development and relapse of MS. Further, we aim to observe the association of adverse early‐life events with the age of disease onset, quality of life and other clinical manifestations in MS individuals. Results from this review would help medical practitioners in identifying individuals at risk for MS and would also help create awareness among the general population regarding the long‐term implications of ACEs.

## 2 METHODS

This systematic review has been reported in concordance with the guidelines provided by Preferred Items for Systematic Review and Meta‐Analysis (PRISMA) (Liberati et al., 2009). The PRISMA checklist is designed and presented in the Supplementary file. This systematic review has been registered with The International Prospective Register of Systematic Reviews, PROSPERO (CRD42022344970).

### 2.1 Data sources and search strategy

**FIGURE 2:**
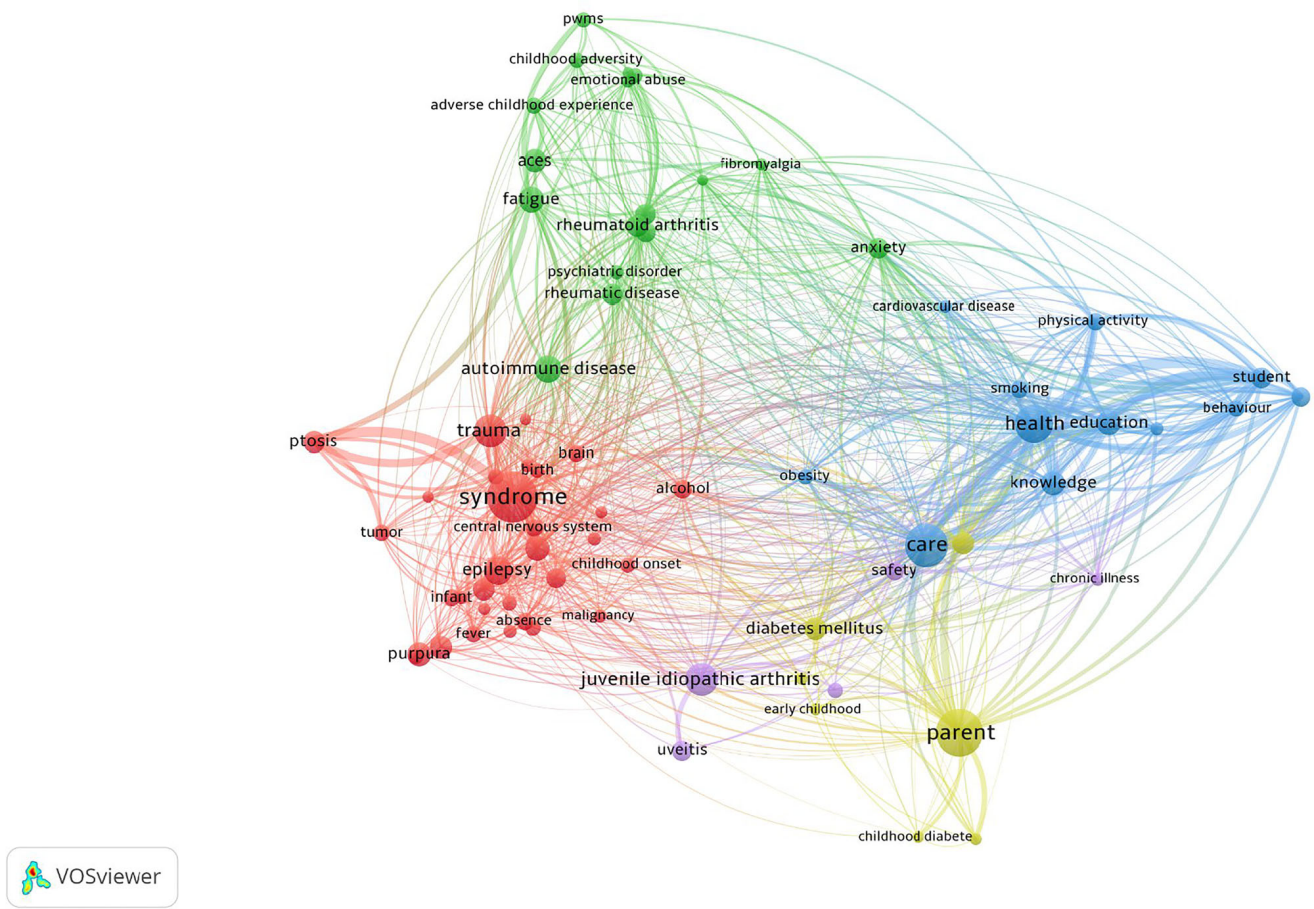
PubMed keyword network map based on occurrence.

A comprehensive electronic search of MEDLINE (via PubMed), Cochrane CENTRAL, ScienceDirect, ERIC, Google Scholar, and Embase (via Ovid) was conducted from January, 1980 till June 18, 2022, by using medical subject headings (MeSH): “childhood experience” OR “adverse childhood experience” OR “childhood trauma” OR “childhood abuse” AND “multiple sclerosis” OR “autoimmune disorder” OR “degenerative disorder” without any time, language or sample size restrictions. The search string was modified and adapted for each database. The complete search strategy used in each of the databases is given in Supplementary Table S1. A relevant keyword network map has been designed by using the software VOSviewer (Figure 2) (www.vosviewer.com). Further, the gray literature, bibliographies, and ancestry search was conducted to recruit additional articles.

### 2.2 Study selection and eligibility criteria

We used the following eligibility criteria to include studies in our review: (1) Population: Adult patients (≥ 18 years) diagnosed with multiple sclerosis or individuals who were exposed to ACEs and were at the risk of developing MS. (2) Exposure: Individuals exposed to adverse childhood experiences (ACEs), which include (a) emotional abuse, physical abuse, sexual abuse, verbal abuse, emotional neglect, and physical neglect, (b) mental illness or substance abuse in the nuclear family, (c) death, remarriage, divorce, or life‐threatening illness of parents, (d) household crowding, the family lost home or victim of a violent crime, and (e) early‐life stress, and sudden shock related to bad news. (3) Comparator: Participants who were not diagnosed with MS, individuals with neurological disorders other than MS, or individuals not exposed to ACEs or no comparators. (4) Outcomes: The primary outcomes of interest are the risk of development of MS, age of disease onset, relapse of disease in individuals exposed to ACE, while the secondary outcomes of interest were the progressive MS subtype, health quality of life, current walking ability, disease severity (assessed via severity of symptoms or premorbid IQ), Fatigue, Coping processes, Alexithymia, Anxiety, perceived social support, emotional well‐being, and functional impairment in patients diagnosed with MS. (5) Study design: Observational studies (cross‐sectional, cohorts, and case controls). Articles that evaluated the association between ACE and other nervous disorders duplicate records and articles in languages other than English were excluded. Case reports, commentaries, and editorials were also excluded. Protocols and articles that were nor peer reviewed were also excluded from our selection.

The articles retrieved from the systematic search were exported to the EndNote Reference Manager (Version X7.5; Clarivate Analytics, Philadelphia, Pennsylvania), where duplicates were located and removed. Two independent reviewers (SHS and ZK) evaluated the rest of the articles based on the title and abstract. The full texts were then evaluated to assess relevance. In case of any disagreement, a third reviewer (STR) was consulted.

### 2.3 Data extraction

Data extraction was completed by two independent reviewers (STR, ZK) by using a self‐designed Microsoft Excel sheet, and discrepancies were resolved through feedback from a third reviewer (SHS). Data on study year, study design, place and duration of the study, sample size, age, gender, type and mode of assessment of ACEs, and age of MS onset were extracted from the eligible articles.

### 2.4 Quality assessment

For cohort and case‐control studies, two investigators (AS and SHS) independently assessed the quality of included studies using the Newcastle‐Ottawa scale (NOS) (Wells et al. 2000). Disagreements on the risk of bias assessments between the two review writers (AS and SHS) were resolved via discussion with a third review author (STR). This scale assigns grades to studies based on three factors (selection, comparability of study groups, and the outcome of interest). A study can receive the highest rating of 9 for cohort and case‐control studies. For cohort and cross‐sectional studies with a total score of 8 or 9 points were deemed to have a low risk of bias; studies with a score of 7 or 6 points were judged to have a moderate risk of bias, and studies with a score of 5 points or less were regarded to have a high risk of bias. Regardless of the quality score, all the articles were included in the review.

### 2.5 Data synthesis

The studies included in this systematic review employed a variety of different statistical methods for analyzing the outcomes. Hence, the outcomes of these articles could not be pooled together for quantitative analysis. We qualitatively synthesized the outcomes, and the study findings are summarized in the results section and tabulated in summary tables.

## 3 RESULTS

### 3.1 Literature review and Study characteristics

**FIGURE 3:**
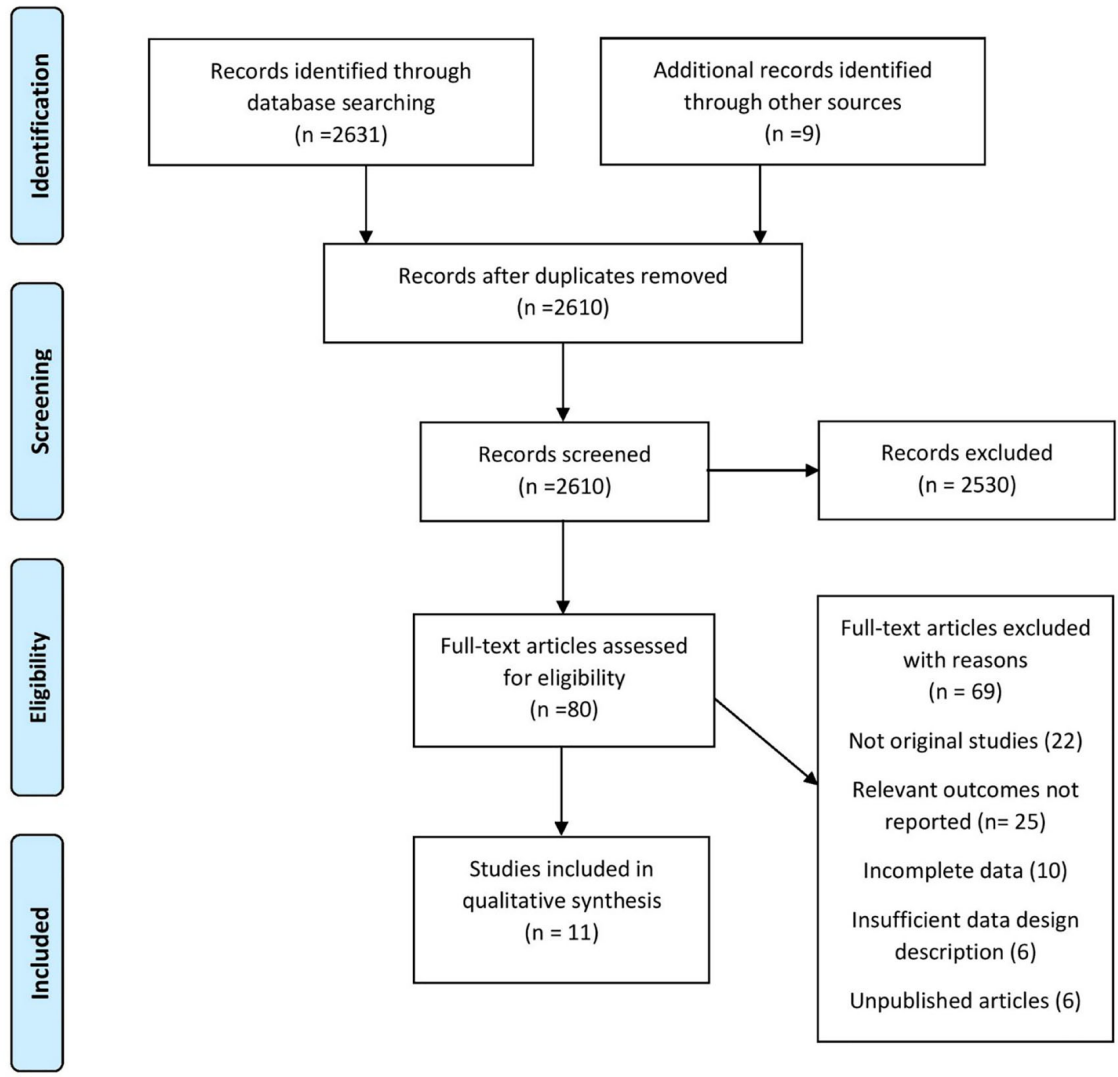
Prisma flow chart of literature search.

**FIGURE 4:**
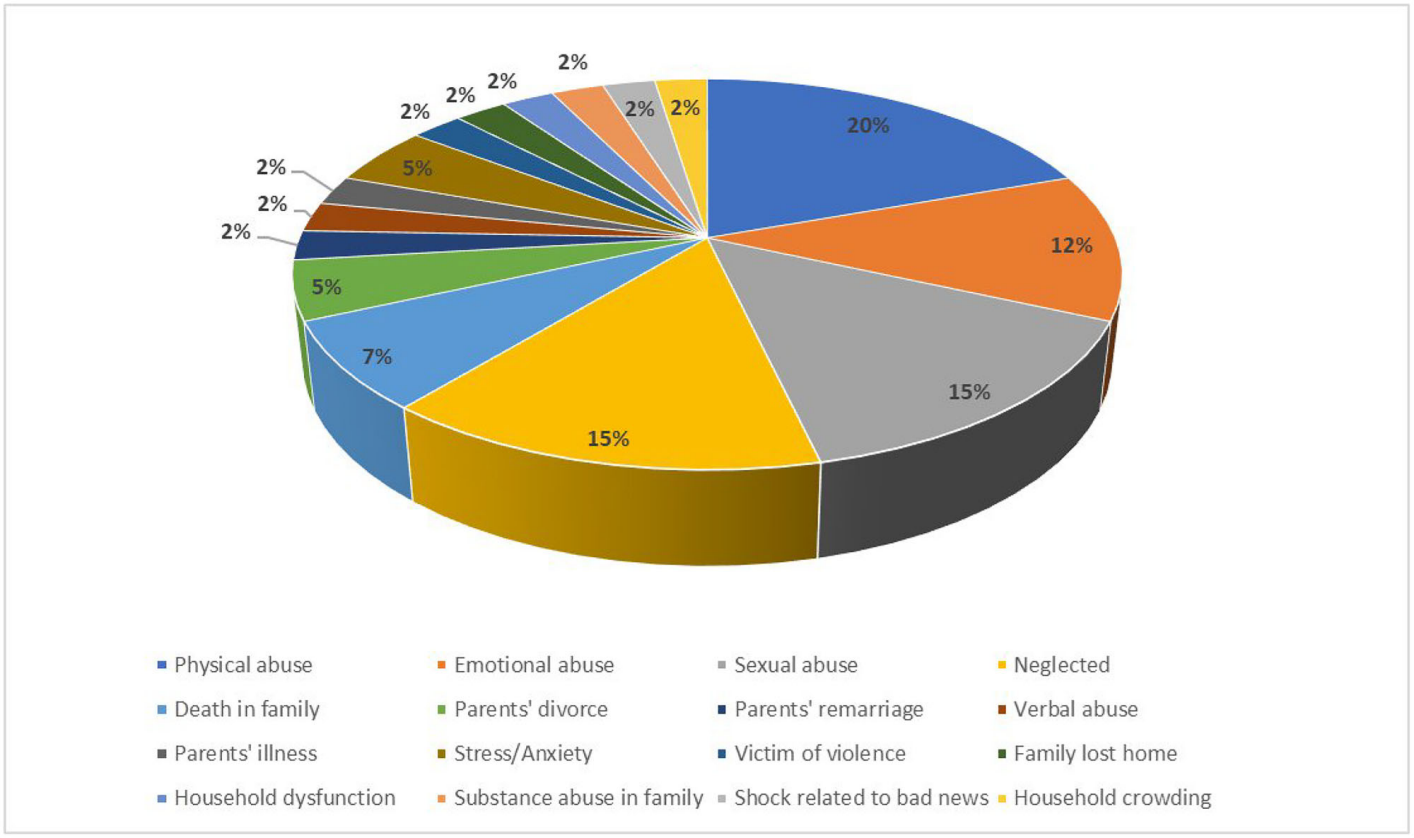
Pie chart depicting percentages of studies assessing different exposures for developing multiple sclerosis.

**FIGURE 5:**
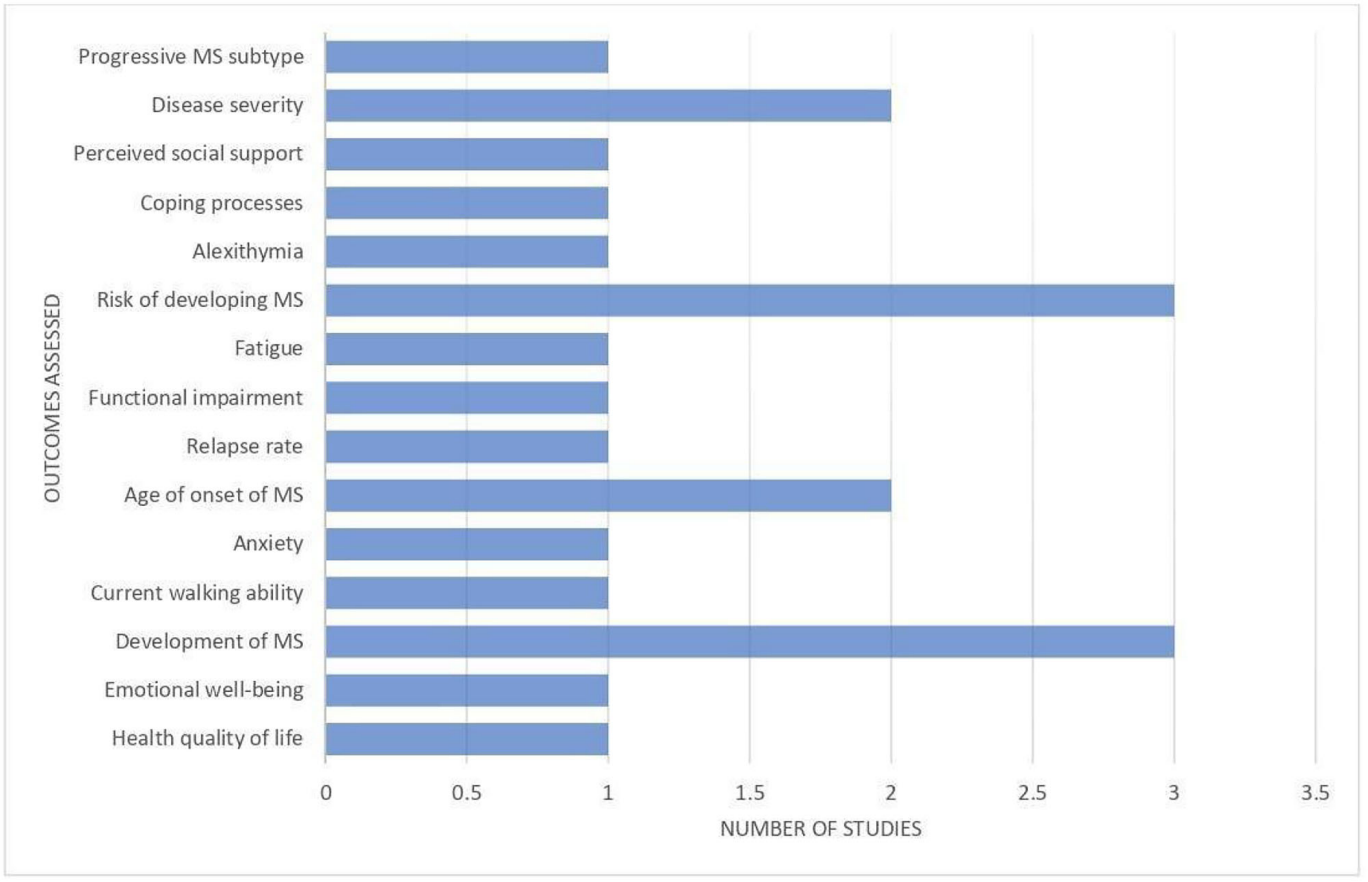
Bar chart representing primary and secondary outcomes assessed by included studies.

The PRISMA flow chart summarizes the search and study selection process (Figure 3). The initial search yielded a total of 2631 potential studies over 6 different electronic databases. After abstract screening and full‐text review, 80 articles were shortlisted. Further, 69 articles were removed that did not meet the inclusion criteria of the review. Eventually, a total of 11 studies were included for qualitative synthesis, which included 6 case‐control studies (Spitzer et al., 2012; Horton et al., 2022; Briones‐Buixassa et al., 2019; Eftekharian et al., 2016; Gunnarsson et al., 2015; Warren et al., 1982), 3 retrospective cohort studies (Shaw et al., 2017; Nielsen et al., 2014; Pust et al., 2020), and 2 prospective cohorts (Eilam‐Stock et al., 2021; Eid et al., 2022). Study characteristics and baseline characteristics of participants are provided in Tables 1 and 2, respectively. Figure 4 depicts the proportion of exposure presented in included studies. A summary of primary and secondary outcomes extracted from each study is presented in Figure 5.

**TABLE 1:** Tabulation of study characteristics and findings

Author (year)	Study design	Place of study	Duration of study	Mode of assessment of ACEs	Mode of MS assessment	Outcomes measured	Findings
Eid et al. (2022)	Prospective cohort	Norway	1999–2008	Self‐administered questionnaires	Revised McDonald Criteria*	MS development	Exposure to emotional abuse, HR 1.40 (95% CI 1.03 to 1.90), physical abuse, HR 1.31(95% CI 0.83 to 2.06), and sexual abuse, HR 1.65 (95%CI 1.13 to 2.39) was positively associated with subsequent MS development. Moreover, when exposed to two (HR 1.66, 95% CI 1.04 to 2.67), or three categories of childhood abuse (HR 1.93, 95% CI 1.02 to 3.67), women were at even more risk of developing MS.
Horton et al. (2022)	Case control	Northern California	2006–2014	Computer‐assisted telephone interview (CATI), 9 ACE questions	Diagnosed by a Neurologist	Age of MS onset Progressive MS subtype (relapsing‐remitting, secondary progressive, primary progressive, or relapsing‐progressive) Current walking ability Disease severity	No significant association between ACE and the risk of MS (OR = 1.01 95% CI: 0.87, 1.18).
Shaw et al. (2017)	Retrospective cohort	Stony Brook, New York	July 2014 to June 2015	10‐item ACE tool	Diagnosed by a Neurologist	Age of MS onset Disease severity via Premorbid IQ (assessed through reading recognition test‐WRAT‐3)	Significant and inverse correlation between ACEs scores and age of MS onset (*r* = −0.30, *p* = .04). Performance on the WRAT‐3 reading recognition was significantly linked to participants’ ACE scores (*r* = −0.25, *p* = .04), indicating that childhood adversity was also predictive of premorbid IQ.
Eilam‐Stock et al. (2021)	Prospective cohort	New York	N/A	10‐item ACE tool	Revised McDonald Criteria	Health‐related quality of life and emotional well‐being in patients diagnosed with MS (assessed via SeMaS Anxiety scale, SeMaS depression scale, and SF‐36 scale	Significant association between ACE scores and SeMaS Anxiety scale (Adjusted *R* ^2^ = 0.12, *F* = 4.29, *p* = .049). However, no significant association was observed between ACEs score and the SF‐36 scale (Adjusted *R* ^2^ = −0.02, *F* = 0.38, *p* = .54) or SeMaS depression scale (Adjusted *R* ^2^ = −0.01, *F* = 0.79, *p* = .38)
Spitzer et al. (2012)	Case control	Germany	N/A	28‐item Childhood Trauma Questionnaire (CTQ)	Revised McDonald Criteria	MS onset Relapse rate Functional impairment	No significant association between severe abuse and MS onset and functional impairment. Physical abuse and emotional neglect were associated with higher relapse rates ((β = 0.22, *p* = .033 and (β = −0.31, *p* = .048, respectively). Severe abuse participants had a significantly higher mean relapse rate of 0.89 (0.68) than the mean relapse rate of 0.62 (0.54) of participants who had no history of severe abuse (*F* = 5.4, *p* = .022, d = 0.44).
Pust et al. (2020)	Retrospective cohort	Germany	July 2018 to March 2019	28‐item Childhood Trauma Questionnaire (CTQ)	Self‐reported diagnosis of MS	Fatigue in MS individuals (assessed via CTQ scale)	A significant association between emotional abuse (*p* < .001), physical abuse (*p* < .01), physical neglect (*p* < .001), emotional neglect (*p* < .001), and fatigue symptoms in MS patients.
Nielsen et al. (2014)	Retrospective cohort	Denmark	1968–2011	N/A	Allison or Poser criteria and McDonald criteria	Risk for MS development	Exposure to a minimum of 1 SFLE has a 1.11 times greater risk for the development of MS (RR = 1.11) ‐ Males (RR = 1.14) and Females (RR = 1.10). Exposure to a single SFLE has an 11% greater risk for MS than NE subjects (RR = 1.11). Exposure to more than one SFLE has a relatively higher risk for MS (RR = 1.17). Exposure to parental divorce has a 13% increased risk for MS (RR = 1.13). Exposure to parental or sibling death does not significantly increase the risk for MS. No significant association between the risk for MS and age at parental divorce (*p* = .68) or age since the parental divorce (*p* = .37) was observed.
Briones‐Buixassa et al. (2019)	Case control	Spain	May 2014 to June 2015	Early‐life stress via CTQ‐SF	McDonald criteria	Coping processes Perceived social support Trait anxiety Alexithymia	No significant association between early‐life stress in pwMS (*p* = .65). When compared to the control group, pwMS reported higher levels of avoidance coping mechanisms (*p* = .003), high trait anxiety (*p* = .002), and alexithymia (*p* = .04). pwMS reported significantly lower levels of perceived social support (*p* = .001).
Eftekharian et al. (2016)	Case control	Hamadan, Iran	N/A	Interview questionnaire	Diagnosed by a neurologist	Risk for MS development	There is a significant association between physical child abuse 2–3 times/week (OR = 18.81 95% CI 4.46–79.38) with increased risk for MS.
Warren et al. (1982)	Case control	Canada	1 Year	Interview questionnaire	Schumacher criteria	Development of MS	There is no significant difference between the controls and pwMS based on the emotional climate of their homes during childhood and adolescence.
Gunnarsson et al. (2015)	Case control	Sweden	1 Jan 1952 to 31 Dec 1956	N/A	ICD, 340 recorded on Swedish NPR	Risk MS development	Exposure to a more crowded household in childhood (4–8 years) is associated with reduced risk for MS (*p* = .007). There is no significant association between parental occupation and risk of MS development (*p* = .438).

MS: multiple sclerosis; E: exposed; NE: not exposed; CTQ‐SF: Childhood Trauma Questionnaire‐Short Form; pwMS: people with multiple sclerosis; ACE: adverse childhood experience; HR: hazard ratio; OR: odds ratio; WRAT‐3: Wide Range Achievement Test‐third edition; IQ: intelligence quotient; SF‐36, Study Short Form–36 Items; SeMaS: Self‐Management Screening; CTQ: Childhood Trauma Questionnaire; OCD: obsessive‐compulsive disorder; SFLE: stressful life event; ICD: International Classification of Diseases; NPR: National Patient Register.

* MS assessment scale details are provided in the Supplementary file.

**TABLE 2:** Tabulation of population characteristics

Author (year)	Number of participants (*N*), sex	Age years; mean (SD), sex	Control group population (*N*) and selection	Types of ACEs	Age of onset of MS years, mean (SD)
Eid et al. (2022)	77,997 Females	Exposed: 29 (5) Unexposed: 30 (5)	63,520 Women not exposed to childhood abuse	Emotional abuse, physical abuse, sexual abuse	Exposed: 33 (6) Unexposed: 33 (7)
Horton et al. (2022)	*N* = 2607 Males with MS (*n* = 298) Females MS (*n* = 1124)	‐	1185 No MS diagnosed Male (*n* = 219) Female (*n* = 966)	Death, remarriage, divorce of parents, the life‐threatening illness of parents. Physical, verbal abuse or neglect. Adopted or lived with other family members. The family lost home, a victim of a violent crime	‐
Shaw et al. (2017)	*N* = 67 Males (*n* = 15) Females (*n* = 52)	50.49 (10.67)	‐	Household dysfunction, Neglect, emotional, physical, and sexual abuse	32.40 (11.67)
Eilam‐Stock et al. (2021)	*N* = 31, Males (*n* = 6) Females (*n* = 25)	33.84	‐	Physical, verbal, or sexual abuse, mental illness, or substance abuse in the nuclear family, measured on a scale of 1–10	‐
Spitzer et al. (2012)	*N* = 1119, Males with MS (*n* = 63) Females with MS (*n* = 171) AGE MS group: 39.7 (7.1)	MS group: 39.7 (7.1) Control group: Age: 41.2 (5.7)	885 Healthy population without cognitive impairment Males (*n* = 373) Females (*n* = 512)	Emotional, sexual, and physical abuse and emotional and physical neglect	29.2 (7.8)
Pust et al. (2020)	*N* = 571 Males (*n* = 133) Females (*n* = 438) AGE: 43.4 (10.9)	43.4 (10.9)	‐	Emotional abuse, physical abuse, sexual abuse, emotional neglect, physical neglect.	‐
Nielsen et al. (2014)	*N* = 3260 NE Males (*n* = 713) E Males (*n* = 276) NE Females (*n* = 1645 NE) E Females (*n* = 626)	‐	‐	Stressful life events: Parental divorce, parental death; death of a sibling	‐
Briones‐Buixassa et al. (2019)	*N* = 41 Males (*n* = 12) Females (*n* = 29)	MS group: 48.48 (11.85) Control: 48.99 (12.02) years	41 Males (*n* = 12) Females (*n* = 29) Age: 48.99 (12.02) years	Early‐life stress: emotional and physical neglect; emotional, sexual, and physical abuse	35.93 (10.71); Range = 12–62)
Eftekharian et al. (2016)	*N* = 250 Males (*n* = 64) Females (*n* = 186)	‐	250 Males (*n* = 69) Females (*n* = 181)	Physical child abuse, head trauma, stress and anxiety disorders, OCD, depression, sudden shock related to bad news	*N*/A
Warren et al. (1982)	*N* = 100, Males (*n* = 30) Females (*n* = 70)	‐	100 Rheumatology patients (*n* = 30) Patients with neurologic conditions except for MS (*n* = 43)	Emotional stress during childhood	66% developed the initial symptoms in 20–39 years
Gunnarsson et al. (2015)	628 Males	‐	Males (*n* = 6187) (1:10 ratio between cases and healthy controls)	Household crowding, parental occupation	‐

MS: multiple sclerosis; E: exposed; NE: not exposed; OCD: obsessive‐compulsive disorder.

### 3.2 Quality assessment

Five cohort studies and six case‐control studies were examined for bias; all 11 cohort and case‐control studies had a low to medium risk of bias. Shaw et al. (2017), Eilam‐Stock et al. (2021), and Pust et al. (2020) did not report the inclusion of an unexposed cohort. Pust et al. (2020) did not mention a follow‐up time for the outcomes to occur. Spitzer et al. (2012) and Horton et al. (2022) did not provide sufficient information about the case and control group response rates. Eftekharian et al. (2016) did not demonstrate a viable method of determining patient exposure. Warren et al. (1982) is the only case‐control study that used a hospital control group. Summary of quality assessment is present in Supplementary Tables S2 and S3.

### 3.3 Study characteristics

The 11 included studies of a total of 86,671 participants were conducted from December 1956 to June 2022, assessing the association between adverse childhood experiences and multiple sclerosis. The scale which assessed the ACEs varied from study to study. The Childhood Trauma Questionnaire (CTQ) measures adversity in childhood including physical and emotional neglect, emotional and physical abuse and sexual abuse. Spitzer et al. (2012), Briones‐Buixassa et al. (2019), and Pust et al. (2020) employed the CTQ to evaluate the severity of ACEs in the patient population. Shaw et al. (2017) and Eilam‐Stock et al. (2021) employed the 10‐item Childhood Adversity Scale to assess adverse childhood experiences. Eid et al. (2022), Horton et al. (2022), Eftekharian et al. (2016), and Warren et al. (1982) evaluated ACEs using interview questionnaires. Gunnarsson et al. (2015) and Nielsen et al. (2014) did not mention a specific assessment tool in the study methods.

### 3.4 Association of adverse childhood experiences with risk of developing multiple sclerosis

Eid et al. (2022) used an interview form that included questions about humiliation, physical abuse, and sexual abuse to assess the relationship between ACEs and the development of MS in pregnant women. When the exposed cohort was compared to the control group, which did not report any kind of childhood abuse, the findings of the cohort study demonstrated a prevalent relationship between childhood maltreatment and the development of MS 23. Following the follow‐up period, 300 women acquired MS, with 71 (24%) reporting a history of childhood abuse (Eid et al., 2022). Further research revealed a strong link between ACEs and the development of MS [Hazards Ratio (HR): 1.31; 95% CI: 0.99, 1.72] (Eid et al., 2022). Emotional and sexual maltreatment were also highly related to an MS diagnosis, with HRs of 1.40 (95% CI: 1.03, 1.90) and 1.65 respectively (95% CI: 1.13, 2.39) (Eid et al., 2022). Horton et al. employed the 10‐item CTQ with exposure to each ACE contributing to the total ACEs score. A score of 4 or more indicated severe childhood experiences (Horton et al., 2022). This cohort study found no significant connection between having ACEs before the onset of MS when comparing the patient cohort to a control group that had no ACEs (OR: 1.01; 95% CI: 0.87, 1.18) (Horton et al., 2022). Abuse and home loss were the only two unfavorable childhood experiences linked to the development of MS (OR: 0.66; 95% CI: 0.52, 0.84) and (OR: 0.61; 95% CI: 0.45, 0.82), respectively (Horton et al., 2022). Only the age group of 0–10 years was determined to be statistically significant (False Discovery Rate) (FDR *q* < .05) (Horton et al., 2022). Using the ACE tool, Shaw et al. (2017) discovered a strong inverse connection between the age of onset of MS and ACEs (*r* = 0.30, *p* = .04), indicating a link between childhood abuse and early onset of MS. Spitzer et al. (2012) employed the 28‐item CTQ for the assessment of adverse childhood experiences, which are rated on a 5‐point scale with a higher score being indicative of greater adverse experiences during childhood to assess the outcome. When compared to the unexposed general population, MS patients had a significantly higher overall CTQ score in this cohort research (*p* = .001) (Spitzer et al., 2012). Further CTQ analysis revealed that emotional abuse (*p* = .001), sexual abuse (*p* = .003), and emotional neglect (*p* = .001) were all substantially related to an MS diagnosis (Spitzer et al., 2012). MS relapse rates were strongly linked with physical maltreatment (β = 0.22, *p* = .033) and emotional neglect (β = −0.31, *p* = .048), respectively (Spitzer et al., 2012).

When the CTQ's short form questionnaire, which includes a score of 0–108; with a higher score indicating greater childhood adversities, was employed to assess our primary outcome, Briones‐Buixassa et al. (2019) found no significant association between early‐life stressful experiences and an MS diagnosis (*p* = .40).

Nielsen et al. (2014) analyzed the association between stressful life experiences (SFLEs) and the onset of MS. SFLEs are characterized as exposure to parental divorce, parental death, or the death of a sibling before the age of 18 years. This study did not use an evaluation method to quantify ACEs, but the study found that having SFLEs before the age of 18 increased the probability of developing MS (RR: 1.11; 95% CI: 1.03, 1.20) (Nielsen et al., 2014). Gunnarsson et al. (2015) used no evaluation tool and found a significant link between higher house crowding and a lower probability of having MS between the ages of 4 and 8 years (*p* = .007).

Warren et al. (1982) did not employ any assessment tool to evaluate the relation between the development of MS and childhood adversities. The participants reported that 17% of them had unhappy home lives in their childhood and adolescence, with 46% of them experiencing moderately happy childhood lives and 35% experiencing very happy home lives during their childhood (Warren et al., 1982).

### 3.5 Association of ACEs with parameters of multiple sclerosis

Eilam‐Stock et al. (2021) investigated the relationship between health‐related quality of life and emotional well‐being in people with MS who had ACEs. A 10‐item validated questionnaire was used to assess the exposure to childhood adversities, including verbal, physical, or emotional abuse (Eilam‐Stock et al., 2021). The Self‐Management Screening (SeMaS) method was used to assess emotional well‐being in MS patients, and the results revealed a strong correlation with ACEs (*r* = 0.39, *p* = .025) (Eilam‐Stock et al., 2021). The higher the prevalence of ACEs, the higher the SeMaS anxiety score. The SF‐36 scale (Study Short Form‐36 Items) (Adjusted *R*2 = 0.02, *F* = 0.38, *p* = .54) and SeMaS Depression scale (Adjusted *R* ^2^ = 0.01, *F* = 0.79, *p* = .38) were not significantly linked with ACEs in the MS cohort (Eilam‐Stock et al., 2021). Shaw et al. (2017) discovered a significant relationship between the Wide Range Achievement Test‐third edition (WRAT‐premorbid IQ3 reading recognition) and ACE scores (*r* = 0.25, *p* = .04), which is indicative of premorbid IQ.

Pust et al. **(2020)** evaluated fatigue symptoms in persons with MS by employing the CTQ assessment tool. The CTQ is a self‐reported assessment tool that assesses emotional, physical, sexual abuse, emotional and physical neglect experienced by children 12 years or older (Pust et al., 2020). The analysis revealed a significant association between emotional abuse (*p* < .001), physical abuse (*p* < .01), physical neglect (*p* < .001), emotional neglect (*p* < .001), and fatigue symptoms which were assessed using the Fatigue Scale for Motor and Cognitive Functions (FSMC) (Pust et al., 2020). Fatigue symptoms were further analyzed by Chalder Fatigue Questionnaire (CFQ) and similar significant correlations were found between emotional abuse (*p* < .001), emotional neglect (*p* < .001), and physical neglect (*p* < .001) (Pust et al., 2020).

### 3.6 Association of the severity of ACEs with multiple sclerosis

Eftekharian et al.’s (2016) study investigated multiple ACEs for a link to the onset of MS. Physical childhood abuse was graded according to the intensity and frequency of the abuse (Eftekharian et al., 2016). Abuse that occurred 2–3 times per week was associated with an 18.81‐fold increased risk of having MS when compared to the unexposed sample (OR: 18.81; 95% CI: 4.46, 79.38) (Eftekharian et al., 2016). Similar patterns were detected for abuse occurring 2–3 times per month and 2–3 times per year (OR: 1.80; 95% CI: 0.30, 2.14) and OR: 1.27; 95% CI: 0.50, 3.18, respectively), but these findings were not significant (OR <1<) (Eftekharian et al., 2016). Similar tendencies in the intensity of ACEs were studied by measuring negative thoughts (Eftekharian et al., 2016). Negative thoughts experienced during childhood frequently increased the likelihood of getting MS compared to negative thoughts occasionally encountered (OR: 4.83; 95% CI: 3.03, 7.71, and OR: 1.74; 95% CI: 1.08, 2.80), respectively (Eftekharian et al., 2016). Eilam‐Stock et al. (2021) investigated the severity of ACEs further. Severe ACEs were linked with higher SeMaS depression scores at follow‐up [*F*(2, 28) = 5.05, *p* = .02]. When compared to low ACEs, high ACEs were substantially associated with greater depressive symptoms (*p* = .02) (Eilam‐Stock et al., 2021). At follow‐up, the SF‐36 score was likewise found to be substantially associated with increased ACEs [*t*(11) = 2.28, *p* = .04] (Eilam‐Stock et al., 2021). Horton et al. (2022) discovered a link between having at least four ACEs and having MS at a younger age (*r* = −1.99, 95% CI: −3.62, −0.37, *p* = .02). Spitzer et al. (2012) investigated the relationship between the mean relapse rate of MS and the severity of ACEs, which was found to be substantially greater in severe cases of ACEs than in MS patients who did not report any history of misuse (*F* = 5.4, *p* = .022, *d* = 0.44).

## 4 DISCUSSION

In this systematic review, we studied the association between ACEs with the development of MS, age of onset, relapse rates, and quality of life in MS patients by using different parameters described previously in the result section. A total of 11 observational studies with 86,671 participants were included in this systematic review. Of the 11 studies, 9 assessed the association between ACEs and the risk of developing MS (Shaw et al., 2017; Spitzer et al., 2012; Eid et al., 2022; Horton et al., 2022; Briones‐Buixassa et al., 2019; Eftekharian et al., 2016; Gunnarsson et al., 2015; Warren et al., 1982; Nielsen et al., 2014). Four studies found a significant association between the development of MS and ACEs (Spitzer et al., 2012; Eid et al., 2022; Eftekharian et al., 2016; Nielsen et al., 2014), as shown in Figure 8, whereas three studies found no significant association between the risk for development of MS and ACEs (Horton et al., 2022; Briones‐Buixassa et al., 2019; Warren et al., 1982). Higher household crowding reduces the risk of the development of MS (Gunnarsson et al., 2015). A study identified an inverse relationship between ACEs and the age of onset of MS (Shaw et al., 2017). Studies included in this review also evaluated the association between ACEs and different clinical parameters of MS. There is a significant association between ACEs with anxiety, use of walking aid, premorbid IQ, and fatigue symptoms in MS patients (Shaw et al., 2017; Eilam‐Stock et al., 2021; Horton et al., 2022; Pust et al., 2020). Four studies assessed the relationship between MS and the severity of ACEs. Increased frequency of physical childhood abuse and negative thoughts are associated with an increased risk of MS. Multiple ACEs are linked with early‐onset MS, whereas the severity of ACEs was found to be proportional to mean relapse rates of MS (Spitzer et al., 2012; Eilam‐Stock et al., 2021; Horton et al., 2022; Eftekharian et al., 2016).

ACEs are characterized by neglect, household dysfunction, physical abuse, emotional abuse, and sexual abuse during childhood. The number of ACEs during childhood is associated with adverse outcomes in MS in a dose–response relationship. Individuals with ACEs are more likely to experience obesity and tobacco use, indirectly contributing to the development and progression of MS (Polick et al., 2022). Exposure to ACEs manifests as functional and structural changes in the brain, such as changes in the cortical volumes, the activation pattern of the brain during stress, and connectivity of white matter (Wan et al., 2022). ACEs also alter glucocorticoid signaling and the hypothalamic‐pituitary‐adrenal axis function, leading to a chronic inflammatory state (Wan et al., 2022). Owing to these functional and structural changes in the brain, ACEs are associated with other neurological disorders, including dementia, Alzheimer's disease, epilepsy, memory impairment, and attention‐deficit/hyperactivity disorder (ADHD) (Corney et al., 2022; Ortiz et al., 2022; Roberts et al., 2022).

**FIGURE 6:**
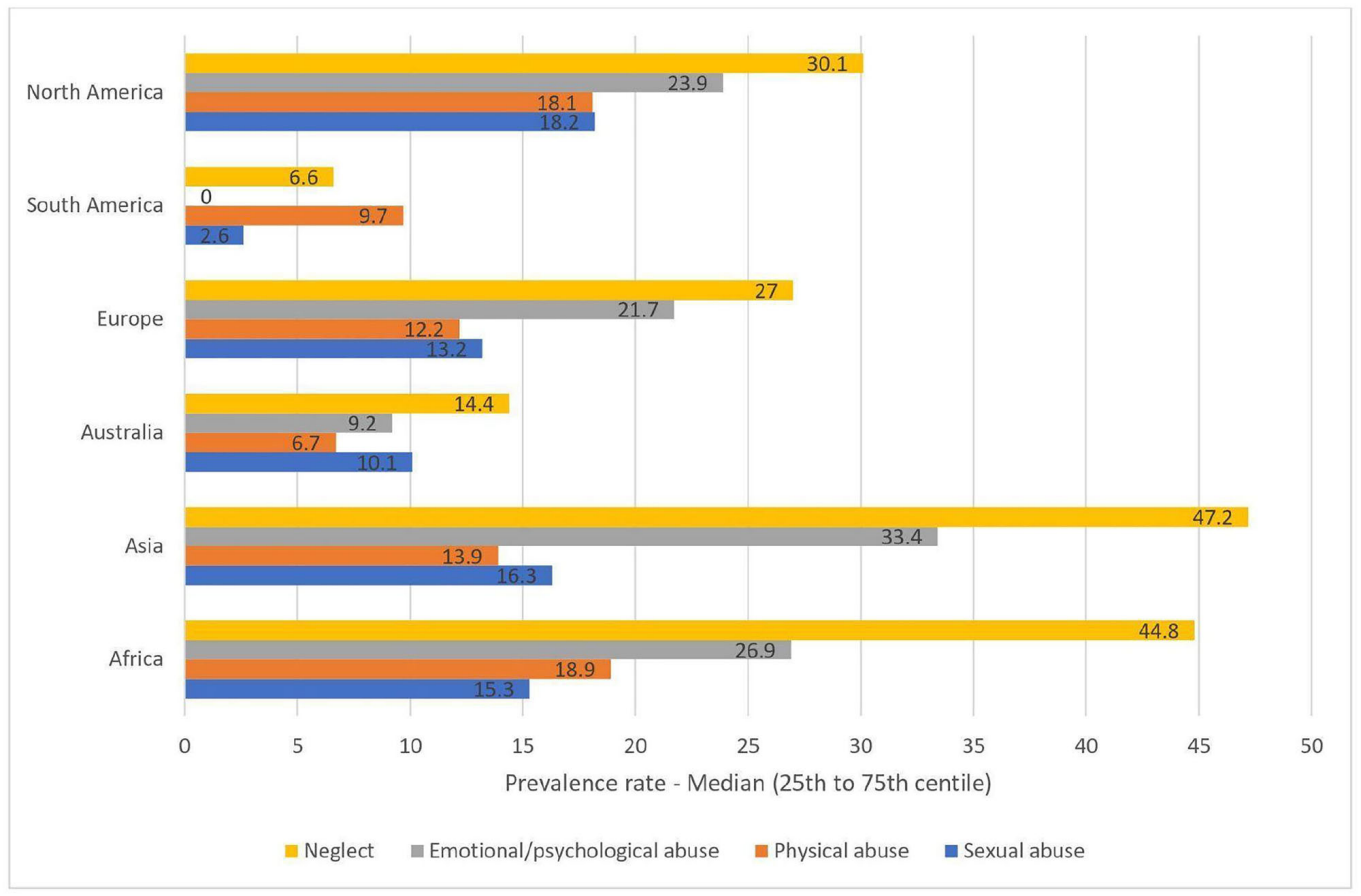
Continent‐wise prevalence rates of multiple ACEs (Moody et al., 2018).

**FIGURE 7:**
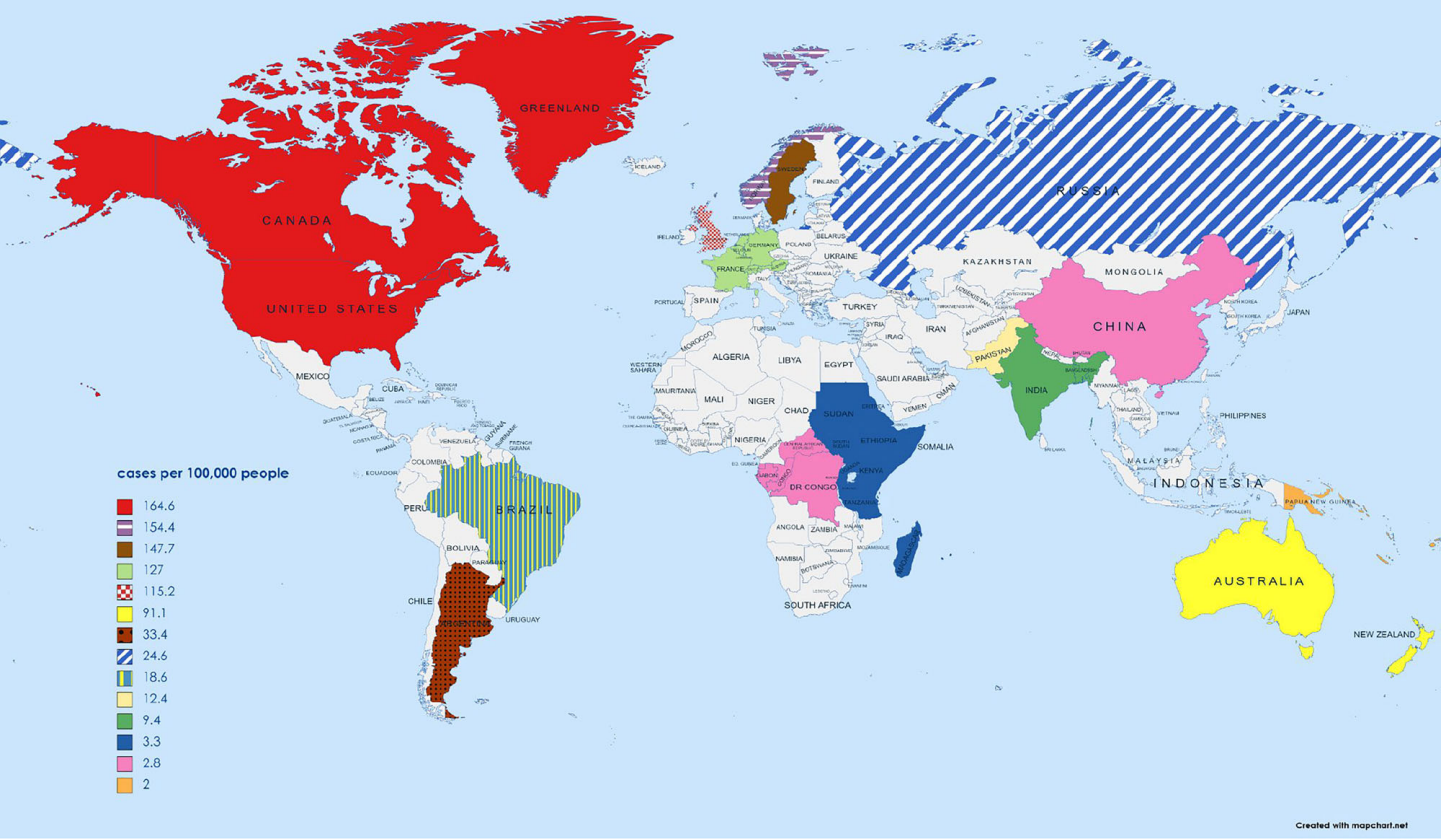
Prevalence of MS per 100,000 people in developed and underdeveloped regions (Wallin et al., 2019; Moghaddam et al., 2021; Gökçe et al., 2019).

According to the literature review, the prevalence of ACEs was variable across different regions of the world. A meta‐analysis conducted by Zhang et al. (2020) revealed that North America accounts for the highest prevalence rates for physical neglect, emotional neglect, physical abuse, and sexual abuse. Sexual abuse, emotional abuse, and neglect are more common among North American girls. A higher prevalence of physical abuse is also observed in Africa (Moody et al., 2018). On the same side, the prevalence of MS is noticed as high as the aforementioned forms of abuse in developed countries, with approximately 5.5 times higher rate of incidence of MS development in developed countries as compared to developing countries (Moghaddam et al., 2021). North America and Europe have the highest prevalence rates for MS, accounting for 140/100,000 and 108/100,000 cases, respectively (Gökçe et al., 2019). The relatively low prevalence rate of MS in Africa can be attributed to a lack of improvement in diagnosis of cases with a mild clinical presentation, decreased awareness of MS, and poor healthcare services 42. The prevalence map of ACEs is presented in Figures 6 and 7 show the prevalence of MS in developed and underdeveloped regions of the world.

**FIGURE 8:**
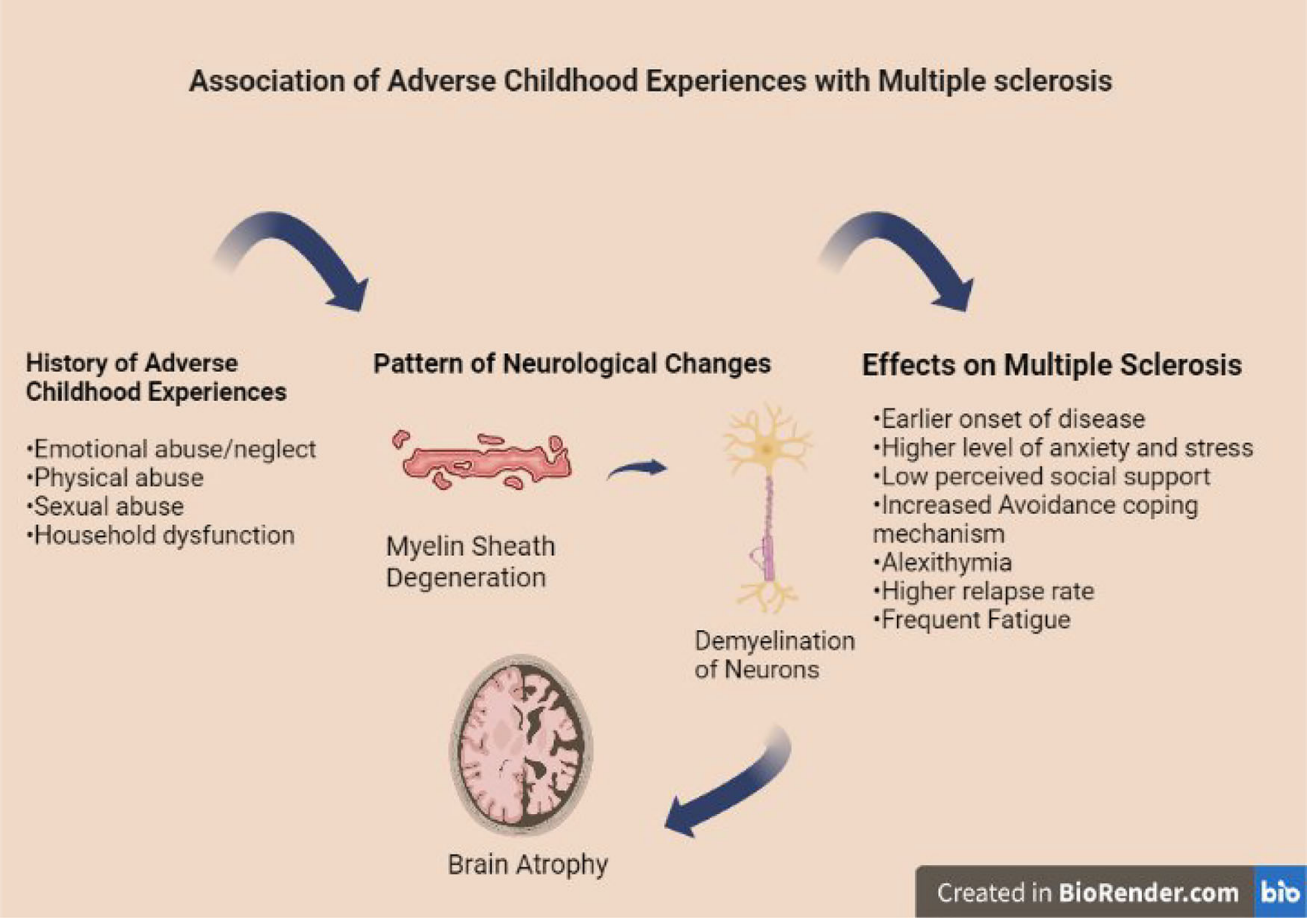
Effects of adverse childhood experiences on multiple sclerosis (BioRender).

ACEs have long‐term effects on physical, mental, and brain health, functioning as major negative stressors. ACEs contribute to higher risks of developing somatic and mental disorders during different stages of life. Disruption in the affective and cognitive processing pathways including increased attention in response to threatening stimuli leads to the development of mental disorders. Depression has the highest risk followed by borderline personality disorder and substance abuse dose‐dependently. The somatic consequences of ACEs include diabetes, abnormal pain perception, obesity, and inflammatory bowel disease. The potential biological mediator between somatic disorders in adulthood and ACEs is innate immune system dysregulation. The profound impact of ACEs on the different aspects of health necessitates the development and implementation of preventive measures from reducing exposure to ACEs and adulthood diseases in the long‐term. (Herzog & Schmahl, 2018)

In addition to the association between the development and parameters of MS and ACEs, prior systematic reviews and meta‐analyses also assessed the relationship between the onset of MS and other childhood experiences. Edwards and Tench (2021) conducted a systematic review and meta‐analysis to investigate the relationship between MS and childhood pet ownership. Factors relevant to the onset of MS include exposure to microorganisms and lack of increased physical activity, and decreased obesity during childhood. However, the authors did not find any significant association between MS and pet ownership or exposure (Edwards & Tench, 2021). In addition to ACEs, physical childhood trauma, premorbid head trauma, in particular, is significantly associated with the risk of developing MS, as demonstrated by Lunny et al. (2014) in their systematic review and meta‐analysis. However, there is no significant association between the onset of MS and spinal injuries, fractures, and burns (Lunny et al., 2014). Another systematic review and meta‐analysis conducted by Lunny et al. (2014) investigated the association between surgery during childhood and the development of MS. The authors found a significant association between childhood appendectomy, tonsillectomy, and the onset of MS. Individuals undergone these surgical procedures were at a greater risk for diagnosis of MS as compared to the control group. The authors did not find any significant relationship between MS diagnosis and other forms of surgeries occurring in childhood (Lunny et al., 2013). A systematic review similar to our study was conducted by Polick et al. (2022) who assessed the relationship between ACEs and MS physical clinical features. Most of the studies included in this review describe the association between ACEs, the prevalence of MS, and the physical clinical features of the disease, including fatigue, pain, disability, age at onset, and relapses . In contrast, we primarily focused on the ACEs and the risk of the development of MS.

There is variability in the relationship between different ACEs and parameters associated with MS. For instance, household crowding has reduced risk for MS (Gunnarsson et al., 2015), as compared to different forms of childhood abuse (Eid et al., 2022). Moreover, in the context of the number of ACEs, children exposed to greater than one SFLEs are at a greater risk for MS. (Nielsen et al., 2014)

This systematic review included 2 prospective cohort studies (Eilam‐Stock et al., 2021; Eid et al., 2022), 3 retrospective studies (Shaw et al., 2017; Nielsen et al., 2014; Pust et al., 2020), and 6 case‐control studies (Spitzer et al., 2012; Horton et al., 2022; Briones‐Buixassa et al., 2019; Eftekharian et al., 2016; Gunnarsson et al., 2015; Warren et al., 1982).  In contrast to case‐control studies that identify the study subjects based on the outcome status, prospective cohort studies identify exposure prior to the outcome and comprise the framework for assessing causality. This allows prospective cohort studies to produce the strongest scientific evidence. However, some of the challenges associated with conducting prospective cohort studies include long follow‐up periods, high rates of loss to follow‐up, and increased expenses. (Song & Chung, 2010) In addition to study design, sample size also influences the study outcomes. While very small samples affect the validity of a research study, large samples may cause clinically insignificant findings to appear as statistically significant differences (Faber & Fonseca, 2014). Even in smaller sample sizes, the precision of study outcomes may improve with longer study durations. Decreased sample size has negative effects on the accuracy of estimated changes and the statistical power of the study. (Feely et al., 2020) Of the studies with known duration of the study, 4 studies had a duration of ≤ 5 years (Shaw et al., 2017; Briones‐Buixassa et al., 2019; Gunnarsson et al., 2015; Pust et al., 2020) and 3 studies had a duration of ≥ 5 years (Eid et al., 2022; Horton et al., 2022; Nielsen et al., 2014). Small sample size associated with the included studies may limit the generalizability of the study outcomes, and ultimately this systematic review (Jiang et al., 2022).

### 4.1 Strengths and limitations

The included studies investigated the association between ACEs, development of MS, and other parameters of MS, which are from diverse regions around the globe. The participants were followed for long periods of time in some studies. Except for a few studies which did not specify specific assessment tools for the measurement of ACEs, the included studies used established questionnaires and assessment tools for measuring ACEs and related outcomes.

There are a few limitations to this review. The first limitation is the observational nature of the studies. There is inconsistency among the included studies concerning the use of assessment tools and the outcomes measured. Few of the included studies fail to report the assessment tool used for reporting ACEs.  In addition, the authors registered the study protocol on July 18, 2022, after the literature search was conducted in June 2022, creating the potential for post hoc changes. However, the authors have disclosed in the protocol registration that they already initiated the process of screening the articles

### 4.2 Future implications

There is an imperative need for prospective studies to assess the relationship between ACEs and MS. Future studies may also investigate the differences in the severity, age‐related onset, and clinical parameters of MS in relation to factors other than childhood adversities as well as compare the outcomes with those of ACEs.

## 5 CONCLUSIONS

In this systematic review of observational studies, the results supported a significant association between ACEs and the development of MS. ACEs also influence the quality of life of MS patients and are associated with the use of walking aid, anxiety, fatigue symptoms, and premorbid IQ. This review also demonstrates that multiple ACEs lead to early‐onset MS and that the severity of ACEs is linked to mean relapse rates of MS. Future studies, preferably randomized controlled trials, shall be conducted to investigate the association between ACEs and MS in large sample sizes.  Offering mental health support and appropriate resources to pwMS may contribute to improved treatment outcomes and health‐related quality of life.

## AUTHOR CONTRIBUTIONS

Conceptualization: STR, HH. Writing—original draft: STR, HH, ZK, SHS, AS, MSA. Review and editing: STR, SS, HAC. Methodology: STR, SHS, SR. Validation: SS, STR, SR. Project administration: STR, HH. Supervision: HAC, SS.

## FUNDING

No financial support was received for the conduct of this study.

## CONFLICT OF INTEREST STATEMENT

The authors declare that they have no conflicts of interest and no financial interests related to the material of this manuscript.

## ETHICS STATEMENT

No ethical approval was required for this study.

## CONSENT

No consent was required for this study.

### PEER REVIEW

The peer review history for this article is available at https://publons.com/publon/10.1002/brb3.3024.

## Supporting information

Figure S1: Prisma checklist for systematic review.
Table S1: Comprehensive search string for each database.
Table S2: Quality assessment for cohort studies.
Table S3: Quality assessment for case‐control studies.

## Data Availability

The data that support the findings of this study are available from the corresponding author upon reasonable request.
