# Supplementary material for: Association of adverse childhood experiences with adulthood multiple sclerosis: A systematic review of observational studies
Source: Brain Behav. 2023 May 1;13(6):e3024. doi: 10.1002/brb3.3024 (PMC10275535; doi:10.1002/brb3.3024)
Supplement: Supplementary file 1 — Figure S1: Prisma checklist for systematic review. Table S1: Comprehensive search string for each database. Table S2: Quality assessment for cohort studies. Table S3: Quality assessment for case‐control studies. [file BRB3-13-e3024-s001.docx]

**Supplementary Appendix**

**Table 1 - Detailed Search Strategy**

| **Database** | **String** | **Results** |
| --- | --- | --- |
| Pubmed | ((("childhood"[All Fields] OR "childhoods"[All Fields]) AND ("experience"[All Fields] OR "experience s"[All Fields] OR "experiences"[All Fields])) OR ("adverse childhood experiences"[MeSH Terms] OR ("adverse"[All Fields] AND "childhood"[All Fields] AND "experiences"[All Fields]) OR "adverse childhood experiences"[All Fields] OR ("adverse"[All Fields] AND "childhood"[All Fields] AND "experience"[All Fields]) OR "adverse childhood experience"[All Fields]) OR ("adverse childhood experiences"[MeSH Terms] OR ("adverse"[All Fields] AND "childhood"[All Fields] AND "experiences"[All Fields]) OR "adverse childhood experiences"[All Fields] OR ("childhood"[All Fields] AND "trauma"[All Fields]) OR "childhood trauma"[All Fields]) OR (("childhood"[All Fields] OR "childhoods"[All Fields]) AND ("abusable"[All Fields] OR "abuse s"[All Fields] OR "abused"[All Fields] OR "abuser"[All Fields] OR "abuser s"[All Fields] OR "abusers"[All Fields] OR "abuses"[All Fields] OR "abusing"[All Fields] OR "abusive"[All Fields] OR "abusively"[All Fields] OR "abusiveness"[All Fields] OR "substance related disorders"[MeSH Terms] OR ("substance related"[All Fields] AND "disorders"[All Fields]) OR "substance related disorders"[All Fields] OR "abuse"[All Fields])) OR ("physical abuse"[MeSH Terms] OR ("physical"[All Fields] AND "abuse"[All Fields]) OR "physical abuse"[All Fields])) AND ("multiple sclerosis"[MeSH Terms] OR ("multiple"[All Fields] AND "sclerosis"[All Fields]) OR "multiple sclerosis"[All Fields] OR ("autoimmune diseases"[MeSH Terms] OR ("autoimmune"[All Fields] AND "diseases"[All Fields]) OR "autoimmune diseases"[All Fields] OR ("autoimmune"[All Fields] AND "disorder"[All Fields]) OR "autoimmune disorder"[All Fields]) OR (("degenerative"[All Fields] OR "degeneratively"[All Fields] OR "degeneratives"[All Fields]) AND ("disease"[MeSH Terms] OR "disease"[All Fields] OR "disorder"[All Fields] OR "disorders"[All Fields] OR "disorder s"[All Fields] OR "disordes"[All Fields]))) | 727 |
| Cochrane CENTRAL | (((childhood experience) OR (adverse childhood experience*) OR (childhood trauma*) OR (childhood abuse*)) AND ((multiple sclerosis*) OR (autoimmune disorder) OR (degenerative disorder))) | 15 |
| Google  Scholar | "childhood experience" OR "adverse childhood experience" AND "Multiple sclerosis" OR "autoimmune disorder" OR "nervous disorder" | 989 |
| Embase | (AllFields:("adverse childhood experience" OR "childhood trauma" OR "childhood abuse") AND ("multiple sclerosis" OR "autoimmune disease")) | 387 |
| Science Direct | ((("childhood experience" OR "adverse childhood experience") AND ("multiple sclerosis" OR "autoimmune disorder" OR "degenerative disorder"))) | 289 |
| ERIC | adverse childhood experience childhood trauma multiple sclerosis autoimmune disorder | 224 |

**Table 2: Newcastle - Ottawa Quality Assessment Scale for cohort studies:**

| **Study** | **Selection** | | | | **Comparability** | **Outcome** | | | **Total^a^** |
| --- | --- | --- | --- | --- | --- | --- | --- | --- | --- |
|  | **1** | **2** | **3** | **4** | **5** | **6** | **7** | **8** |  |
| Eid 2022 | ★ | ★ | ★ | ★ | ★ | ★ | ★ | ★ | 8 |
| Shaw 2017 | ★ | - | ★ | ★ | ★ | ★ | ★ | ★ | 7 |
| Eilam-Stock 2021 | ★ | - | ★ | ★ | ★ | ★ | ★ | ★ | 7 |
| Pust 2022 | ★ | - | ★ | ★ | ★ | ★ | - | ★ | 6 |
| Nielsen 2014 | ★ | ★ | ★ | ★ | ★ | ★ | ★ | ★ | 8 |

**a= out of a maximum score of 9**

**Table 3: Newcastle - Ottawa Quality Assessment Scale for case-control studies:**

| **Study** | **Selection** | | | | **Comparability** | **Exposure** | | | **Total^a^** |
| --- | --- | --- | --- | --- | --- | --- | --- | --- | --- |
|  | **1** | **2** | **3** | **4** | **1** | **1** | **2** | **3** |  |
| Horton 2022 | ★ | ★ | ★ | ★ | ★ | ★ | ★ | **-** | 7 |
| Carsten 2012 | ★ | ★ | ★ | ★ | ★ | ★ | ★ | **-** | 7 |
| Briones-Buixassa 2017 | ★ | ★ | ★ | ★ | ★ | ★ | ★ | ★ | 8 |
| Eftekharian 2017 | ★ | ★ | ★ | ★ | ★ | - | ★ | ★ | 7 |
| Warren 1982 | ★ | ★ | - | ★ | ★ | ★ | ★ | ★ | 7 |
| Gunnarson 2015 | ★ | ★ | ★ | ★ | ★ | ★ | ★ | ★ | 8 |

**a= Out of a maximum score of 9**

Figure 1: Prisma Checklist

| **Section and Topic** | **Item #** | **Checklist item** | **Page Number where item is reported** |
| --- | --- | --- | --- |
| **TITLE** | | |  |
| Title | 1 | Identify the report as a systematic review. | 1 |
| **ABSTRACT** | | |  |
| Abstract | 2 | See the PRISMA 2020 for Abstracts checklist. | 2-3 |
| **INTRODUCTION** | | |  |
| Rationale | 3 | Describe the rationale for the review in the context of existing knowledge. | 4-6 |
| Objectives | 4 | Provide an explicit statement of the objective(s) or question(s) the review addresses. |  |
| **METHODS** | | |  |
| Eligibility criteria | 5 | Specify the inclusion and exclusion criteria for the review and how studies were grouped for the syntheses. | 7-12 |
| Information sources | 6 | Specify all databases, registers, websites, organisations, reference lists and other sources searched or consulted to identify studies. Specify the date when each source was last searched or consulted. | 7-12 |
| Search strategy | 7 | Present the full search strategies for all databases, registers and websites, including any filters and limits used. | 7-12 |
| Selection process | 8 | Specify the methods used to decide whether a study met the inclusion criteria of the review, including how many reviewers screened each record and each report retrieved, whether they worked independently, and if applicable, details of automation tools used in the process. | 7-12 |
| Data collection process | 9 | Specify the methods used to collect data from reports, including how many reviewers collected data from each report, whether they worked independently, any processes for obtaining or confirming data from study investigators, and if applicable, details of automation tools used in the process. | 7-12 |
| Data items | 10a | List and define all outcomes for which data were sought. Specify whether all results that were compatible with each outcome domain in each study were sought (e.g. for all measures, time points, analyses), and if not, the methods used to decide which results to collect. | 7-12 |
|  | 10b | List and define all other variables for which data were sought (e.g. participant and intervention characteristics, funding sources). Describe any assumptions made about any missing or unclear information. | 7-12 |
| Study risk of bias assessment | 11 | Specify the methods used to assess risk of bias in the included studies, including details of the tool(s) used, how many reviewers assessed each study and whether they worked independently, and if applicable, details of automation tools used in the process. | 7-12 |
| Effect measures | 12 | Specify for each outcome the effect measure(s) (e.g. risk ratio, mean difference) used in the synthesis or presentation of results. | N/A |
| Synthesis methods | 13a | Describe the processes used to decide which studies were eligible for each synthesis (e.g. tabulating the study intervention characteristics and comparing against the planned groups for each synthesis (item #5)). | N/A |
|  | 13b | Describe any methods required to prepare the data for presentation or synthesis, such as handling of missing summary statistics, or data conversions. | N/A |
|  | 13c | Describe any methods used to tabulate or visually display results of individual studies and syntheses. | N/A |
|  | 13d | Describe any methods used to synthesize results and provide a rationale for the choice(s). If meta-analysis was performed, describe the model(s), method(s) to identify the presence and extent of statistical heterogeneity, and software package(s) used. | N/A |
|  | 13e | Describe any methods used to explore possible causes of heterogeneity among study results (e.g. subgroup analysis, meta-regression). | N/A |
|  | 13f | Describe any sensitivity analyses conducted to assess robustness of the synthesized results. | N/A |
| Reporting bias assessment | 14 | Describe any methods used to assess risk of bias due to missing results in a synthesis (arising from reporting biases). | N/A |
| Certainty assessment | 15 | Describe any methods used to assess certainty (or confidence) in the body of evidence for an outcome. | N/A |
| **RESULTS** | | |  |
| Study selection | 16a | Describe the results of the search and selection process, from the number of records identified in the search to the number of studies included in the review, ideally using a flow diagram. | 13, FIG5 |
|  | 16b | Cite studies that might appear to meet the inclusion criteria, but which were excluded, and explain why they were excluded. | 13, FIG5 |
| Study characteristics | 17 | Cite each included study and present its characteristics. | 14 |
| Risk of bias in studies | 18 | Present assessments of risk of bias for each included study. | 13-14 |
| Results of individual studies | 19 | For all outcomes, present, for each study: (a) summary statistics for each group (where appropriate) and (b) an effect estimate and its precision (e.g. confidence/credible interval), ideally using structured tables or plots. | N/A |
| Results of syntheses | 20a | For each synthesis, briefly summarise the characteristics and risk of bias among contributing studies. | N/A |
|  | 20b | Present results of all statistical syntheses conducted. If meta-analysis was done, present for each the summary estimate and its precision (e.g. confidence/credible interval) and measures of statistical heterogeneity. If comparing groups, describe the direction of the effect. | N/A |
|  | 20c | Present results of all investigations of possible causes of heterogeneity among study results. | N/A |
|  | 20d | Present results of all sensitivity analyses conducted to assess the robustness of the synthesized results. | N/A |
| Reporting biases | 21 | Present assessments of risk of bias due to missing results (arising from reporting biases) for each synthesis assessed. | N/A |
| Certainty of evidence | 22 | Present assessments of certainty (or confidence) in the body of evidence for each outcome assessed. | N/A |
| **DISCUSSION** | | |  |
| Discussion | 23a | Provide a general interpretation of the results in the context of other evidence. | 28-32 |
|  | 23b | Discuss any limitations of the evidence included in the review. | 32 |
|  | 23c | Discuss any limitations of the review processes used. | 32 |
|  | 23d | Discuss implications of the results for practice, policy, and future research. | 32-33 |
| **OTHER INFORMATION** | | |  |
| Registration and protocol | 24a | Provide registration information for the review, including register name and registration number, or state that the review was not registered. | Prospero [Registration ID: CRD42022344970]. |
|  | 24b | Indicate where the review protocol can be accessed, or state that a protocol was not prepared. | Not Prepared |
|  | 24c | Describe and explain any amendments to information provided at registration or in the protocol. | N/A |
| Support | 25 | Describe sources of financial or non-financial support for the review, and the role of the funders or sponsors in the review. | N/A |
| Competing interests | 26 | Declare any competing interests of review authors. | N/A |
| Availability of data, code and other materials | 27 | Report which of the following are publicly available and where they can be found: template data collection forms; data extracted from included studies; data used for all analyses; analytic code; any other materials used in the review. | N/A |

**Diagnostic criteria for assessment of MS**

- **Schumacher criteria** were the first internationally recognized diagnostic [criteria](https://en.wikipedia.org/wiki/Diagnostic_criteria) that were previously used for identifying [multiple sclerosis](https://en.wikipedia.org/wiki/Multiple_sclerosis) (MS).

To get a diagnosis of MS a patient must show the following:

1. Clinical signs of a problem in the CNS
2. Dissemination in space, shown by clinical evidence of damage in two or more areas of CNS.
3. Evidence of white matter involvement
4. Dissemination in time shown by one of these: Two or more relapses (each lasting ≥ 24 hr and separated by at least 1 month) or disability progression (slow or stepwise)
5. Patient should be between 10 and 50 yr old at time of examination
6. No better explanation for patient’s symptoms and signs should exist

- **Poser criteria** are [diagnostic criteria](https://en.wikipedia.org/wiki/Diagnostic_criteria) for [multiple sclerosis](https://en.wikipedia.org/wiki/Multiple_sclerosis) (MS) which replaced the older [Schumacher criteria](https://en.wikipedia.org/wiki/Schumacher_criteria).

The important aspects for diagnosis of MS are:

1. Attack: Occurrence of a symptom of neurological dysfunction for more than 24 hours
2. Clinical evidence: Neurological dysfunction demonstrable by neurological examination
3. Paraclinical evidence: Demonstration by any test of the existence of a non-clinical lesion in the CNS.

- **McDonald criteria** are the gold standard [diagnostic criteria](https://en.wikipedia.org/wiki/Clinical_case_definition) for [multiple sclerosis](https://en.wikipedia.org/wiki/Multiple_sclerosis) (MS). These new criteria were intended to replace the [Poser criteria](https://en.wikipedia.org/wiki/Poser_criteria) and the older [Schumacher criteria](https://en.wikipedia.org/wiki/Schumacher_criteria). They have undergone revisions in 2005, 2010 and 2017.

The 2017 revision tries to accelerate the diagnosis without risking specificity. The new recommendations include:

1. First of all, probably the most polemical change, a patient with CIS (only one demyelinating lesion) can now be diagnosed as MS if an MRI shows dissemination in space (DIS). In these cases dissemination in time (DIT) can be substituted by a laboratory testing of [oligoclonal bands](https://en.wikipedia.org/wiki/Oligoclonal_band).
2. Second, both symptomatic and asymptomatic lesions can be considered for showing DIS and DIT
3. Third, cortical lesions can also be used to show DIS.
4. Fourth, also for PPMS cortical and asymptomatic lesions can be used in diagnosis.
